# Thyroid Function in Preterm/Low Birth Weight Infants: Impact on Diagnosis and Management of Thyroid Dysfunction

**DOI:** 10.3389/fendo.2021.666207

**Published:** 2021-06-15

**Authors:** Stephen H. LaFranchi

**Affiliations:** Department of Pediatrics, Doernbecher Children’s Hospital, Oregon Health & Sciences University, Portland, OR, United States

**Keywords:** thyroid function, preterm, low birth weight, newborn screening, congenital hypothyroidism, iodine

## Abstract

Maternal thyroid hormone crosses the placenta to the fetus beginning in the first trimester, likely playing an important role in fetal development. The fetal thyroid gland begins to produce thyroid hormone in the second trimester, with fetal serum T4 levels gradually rising to term. Full maturation of the hypothalamic-pituitary-thyroid (HPT) axis does not occur until term gestation or the early neonatal period. Postnatal thyroid function in preterm babies is qualitatively similar to term infants, but the TSH surge is reduced, with a corresponding decrease in the rise in T4 and T3 levels. Serum T4 levels are reduced in proportion to the degree of prematurity, representing both loss of the maternal contribution and immaturity of the HPT axis. Other factors, such as neonatal drugs, e.g., dopamine, and non-thyroidal illness syndrome (NTIS) related to co-morbidities contribute to the “hypothyroxinemia of prematurity”. Iodine, both deficiency and excess, may impact thyroid function in infants born preterm. Overall, the incidence of permanent congenital hypothyroidism in preterm infants appears to be similar to term infants. However, in newborn screening (NBS) that employ a total T4-reflex TSH test approach, a higher proportion of preterm babies will have a T4 below the cutoff, associated with a non-elevated TSH level. In NBS programs with a primary TSH test combined with serial testing, there is a relatively high incidence of “delayed TSH elevation” in preterm neonates. On follow-up, the majority of these cases have transient hypothyroidism. Preterm/LBW infants have many clinical manifestations that might be ascribed to hypothyroidism. The question then arises whether the hypothyroxinemia of prematurity, with thyroid function tests compatible with either non-thyroidal illness syndrome or central hypothyroidism, is a physiologic or pathologic process. In particular, does hypothyroxinemia contribute to the neurodevelopmental impairment common to preterm infants? Results from multiple studies are mixed, with some randomized controlled trials in the most preterm infants born <28 weeks gestation appearing to show benefit. This review will summarize fetal and neonatal thyroid physiology, thyroid disorders specific to preterm/LBW infants and their impact on NBS for congenital hypothyroidism, examine treatment studies, and finish with comments on unresolved questions and areas of controversy.

## Introduction

Thyroid hormone plays a significant role in development and function of every organ system in the body. The presence of triiodothyronine (T3) occupied nuclear receptors in the fetal brain demonstrated by 10 weeks of gestation provides evidence that thyroid hormone has a critical role in brain development (1). Infants born preterm have lower serum thyroid hormone levels as compared to term infants, a reflection of reduced thyroid stimulating hormone (TSH) surge following birth, immature postnatal pituitary-thyroid function, and loss of the maternal contribution. Preterm/low birth weight (LBW) infants are at increased risk for co-morbidities, leading to changes in serum thyroid hormone levels typical of non-thyroidal illness syndrome (NTIS), but which may be difficult to separate from central hypothyroidism. To properly interpret thyroid function tests, newborn screening programs need to be cognizant of these perturbations in T4 and TSH levels in preterm/LBW infants. Clinicians who are consulted for evaluation of potential thyroid dysfunction need to be aware of these dynamic changes to make appropriate management decisions. This review will describe fetal and neonatal thyroid physiology, thyroid dysfunction specific to preterm/LBW infants and their impact on newborn screening (NBS) for congenital hypothyroidism, examine treatment studies, and finish with comments on unresolved questions and areas of controversy.

## Maternal-Fetal Thyroid Relationships

Evidence supports the notion that thyroid hormone present in the fetus in the first trimester is the result of transplacental transfer of maternal hormone (2). This is primarily T4; fetal T3 levels are low as a result of increased placental deiodinase type 3 activity which converts T4 to reverse T3 (RT3). At term, approximately one-third to one-half of cord blood T4 levels are of maternal origin (3). Increased maternal iodine intake during pregnancy is required to keep both mother and fetus iodine sufficient. The recommended dietary allowance (RDA) increases from 150 mcg daily to 250-300 mcg daily with pregnancy (4). As a subgroup, women of reproductive age in the U.S. are borderline iodine deficient (5). Insufficient maternal iodine intake may be a significant factor contributing to lower serum thyroid hormone levels in preterm infants. Maternal hypothyroidism (and maternal iodine deficiency) also are risk factors for preterm birth (6). In addition, excess iodine exposure during pregnancy may also be associated with neonatal hypothyroidism (the Wolff-Chaikoff effect, resulting in temporary decreased production of thyroid hormone). This is a particular problem for preterm/LBW infants, as they are slow to “escape” from this down regulation and may not recover to normal thyroid hormone production for several weeks. Other drug treatment during pregnancy may also affect neonatal thyroid hormone levels. Treatment with steroids or dopaminergic drugs may decrease TSH secretion, resulting in lower neonatal T4 levels.

## Maturation of Fetal Thyroid Function

The thyroid gland starts development at the foramen cecum, migrating to its normal location over the thyroid cartilage. The bilobed thyroid shape is evident by 7 weeks gestation, and thyroid follicles containing colloid are seen histologically by 10 weeks. Thyroglobulin synthesis is detected by 4 weeks, iodine trapping by 8 to 10 weeks, and T4 production by 12 weeks. Significant production of thyroid hormone does not begin until the second trimester (7). Fetal serum T4 concentrations rise from approximately 2 ug/dl (26 nmol/L) at 12 weeks to 10 ug/dL (128 nmol/L) at term (8, 9). Parallel changes are seen in fetal serum free T4 concentrations, rising from approximately 0.1 ng/dL (1.3 pmol/L) at 12 weeks to 2 ng/dL (25.7 pmol/L) at term. As noted above, fetal serum T3 concentrations are low as compared to infant levels, rising from approximately 6 ng/dL (0.09 nmol/L) at 12 weeks to 45 ng/dL (0.68 nmol/L) at term. Thyrotropin releasing hormone (TRH) is present in hypothalamic neurons by 6-8 weeks gestation, and TSH secretion can be detected by 12 weeks. Fetal serum TSH concentrations rise from approximately 4 mIU/L at 12 weeks gestation to 8 mIU/L at term (8, 9). Maturation of the hypothalamic-pituitary-thyroid axis occurs in the second half of gestation, but normal feedback relationships are not mature until term gestation.

## Postnatal Thyroid Function in Preterm/Low Birth Weight Infants

**Figure 1:**
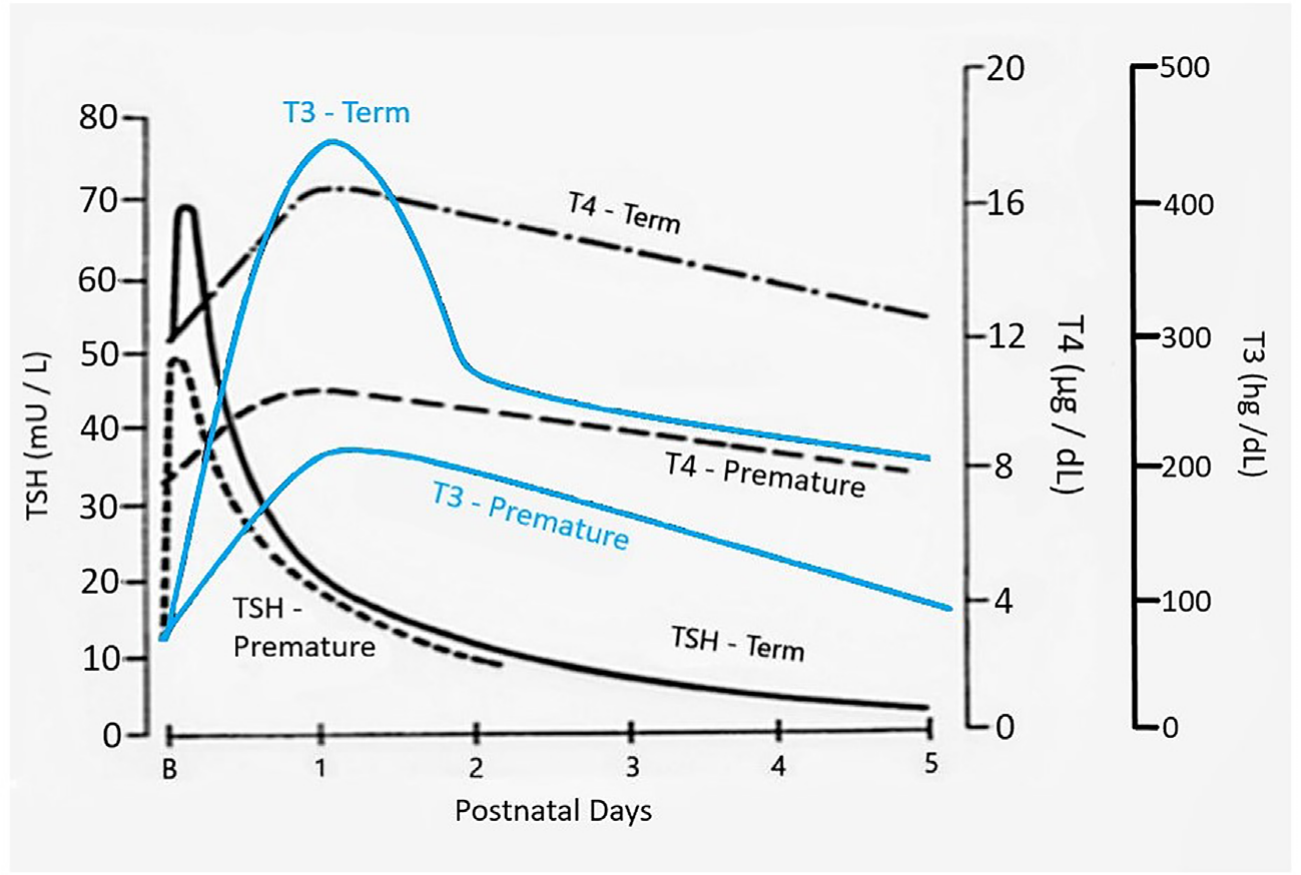
Changes in serum TSH, T4, and T3 following birth in term as compared to preterm newborn infants. Figure developed (with permission) from Fisher DA Thyroid system immaturities in very low birth weight premature infants. Semin Perinatol 2008; 32:387-397.

**Figure 2:**
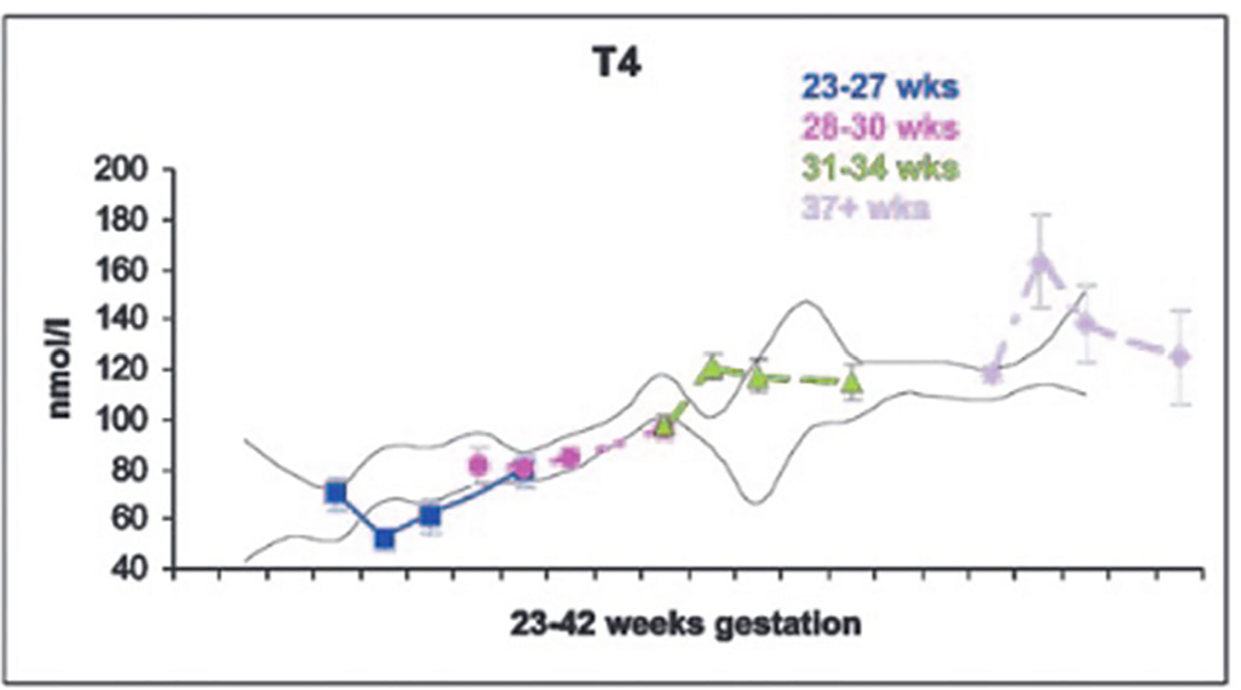
Serum T4 levels in cord blood, and at 7, 14 and 28 days of life in neonates of 23-27, 28-30, 31-34, and 37+ weeks gestation. With permission from Williams FL, Simpson J, Delahunty C, et al. Developmental trends in cord and postpartum serum thyroid hormones in preterm infants. J Clin Endocrinol Metab 2004; 89:5314-5320 (9).

**Figure 3:**
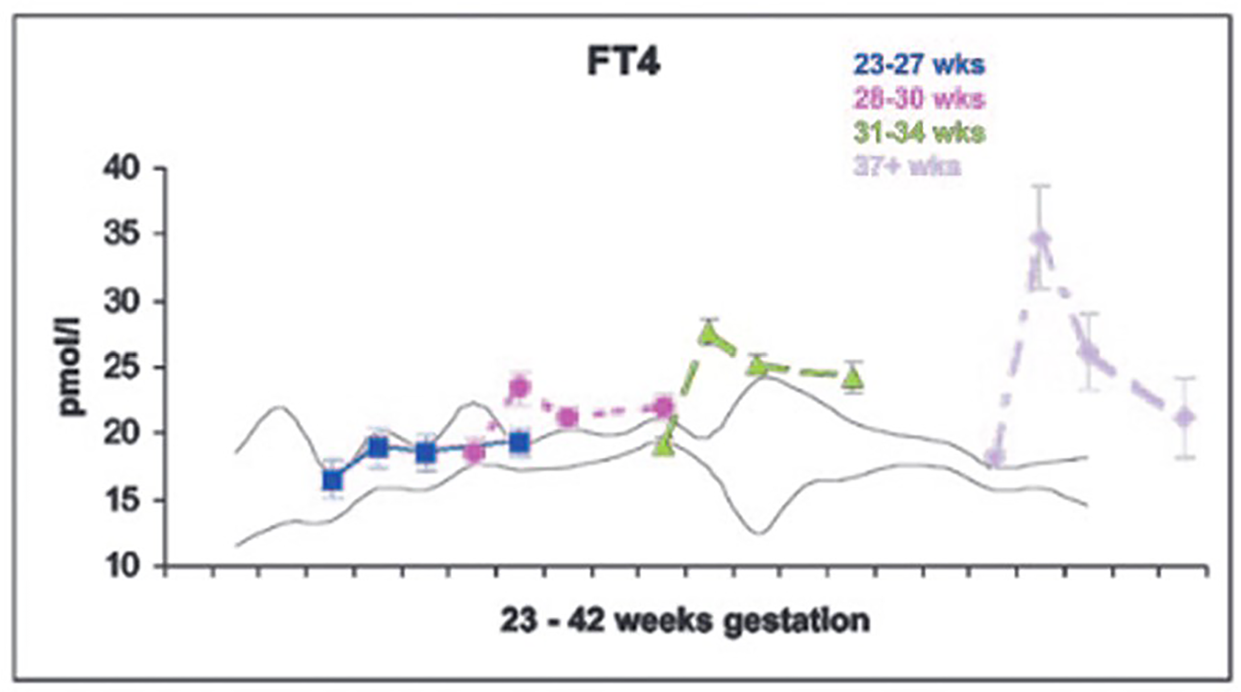
Serum free T4 levels in cord blood, and at 7, 14 and 28 days of life in neonates of 23-27, 28-30, 31-34, and 37+ weeks gestation. With permission from Williams FL, Simpson J, Delahunty C, et al. Developmental trends in cord and postpartum serum thyroid hormones in preterm infants. J Clin Endocrinol Metab 2004; 89:5314-5320 (9).

The dramatic rise in serum TSH 30 to 60 minutes following delivery is reduced in preterm infants as compared to term infants, generally in proportion to their degree of prematurity (10). The peak approximates 30 to 50 mIU/L in preterm infants vs. 60 to 80 mIU/mL in term infants (**Figure 1**). Cord blood T4 levels are lower in infants born preterm, again generally in proportion to the degree of prematurity (**Table 1**). In response to the dramatic TSH rise, serum T4 and T3 levels are elevated at 24 hours following birth in term infants, gradually falling back to “normal” infant ranges around 5 to 7 days of age. In contrast, in the most preterm infants born at 23 to 27 weeks gestation, serum total T4 levels actually decrease in the first week of life, are “level” in infants born at 28 to 30 weeks gestation, while only those babies born >30 weeks gestation show a rise in total T4 in the first week (**Figure 2**). The differences in total T4 concentrations in term *vs.* preterm babies is explained by a combination of immature thyroid hormone production from the thyroid gland, decreased binding proteins (primarily thyroxine binding globulin [TBG]) by the liver, and the effects of NTIS. Postnatal serum free T4 levels are also lower in the first weeks of life, though proportionally not as low as total T4 levels (**Table 1**, **Figure 3**). Serum total T3 levels follow a pattern similar to total T4, though the levels are proportionally even lower in the most preterm babies, likely a reflection of the impact of NTIS (sometimes referred to as the “low T3 syndrome”) (**Table 1**). Administration of certain drugs used to manage co-morbidities in preterm infants may affect thyroid hormone levels; glucocorticoids and dopaminergic agents may inhibit TSH secretion, while excess iodine may inhibit thyroid hormone production.

**Table 1:** Concentrations of free T4, T4, T3, and TSH in preterm and term infants in cord blood at birth, and at 7, 14, and 28 days of age (mean +/- 1 SD).

Gestation (weeks)	Age of Infant	Free T4 (ng/dL)	T4 (μg/dL)	T3 (ng/dL)	TSH (mU/L)
23-27 weeks	Cord	1.28 ± 0.4	5.4 ± 2.0	20 ± 15	6.8 ± 2.9
	7d	1.47 ± 0.6	4.0 ± 1.8	33 ± 20	3.5 ± 2.6
	14d	1.45 ± 0.5	4.7 ± 2.6	41 ± 25	3.9 ± 2.7
	28d	1.50 ± 0.4	6.1 ± 2.3	63 ± 27	3.8 ± 4.7
28-30 weeks	Cord	1.45 ± 0.4	6.3 ± 2.0	29 ± 21	7.0 ± 3.7
	7d	1.82 ± 0.7	6.3 ± 2.1	56 ± 24	3.6 ± 2.5
	14d	1.65 ± 0.4	6.6 ± 2.3	72 ± 28	4.9 ± 11.2
	28d	1.71 ± 0.4	7.5 ± 2.3	87 ± 31	3.6 ± 2.5
31-34 weeks	Cord	1.49 ± 0.3	7.6 ± 2.3	35 ± 23	7.9 ± 5.2
	7d	2.14 ± 0.6	9.4 ± 3.4	92 ± 36	3.6 ± 4.8
	14d	1.98 ± 0.4	9.1 ± 3.6	110 ± 41	3.8 ± 9.3
	28d	1.88 ± 0.5	8.9 ± 3.0	120 ± 40	3.5 ± 3.4
≥37 weeks	Cord	1.41 ± 0.3	9.2 ± 1.9	60 ± 35	6.7 ± 4.8
	7d	2.70 ± 0.6	12.7 ± 2.9	148 ± 50	2.6 ± 1.8
	14d	2.03 ± 0.3	10.7 ± 1.4	167 ± 31	2.5 ± 2.0
	28d	1.65 ± 0.3	9.7 ± 2.2	176 ± 32	1.8 ± 0.9

T4, thyroxine; T3, triiodothyronine; TSH, thyroid stimulating hormone.

Adapted with permission from: Williams FL, Simpson J, Delahunty C, et al. Developmental trends in cord and postpartum serum thyroid hormones in preterm infants. J Clin Endocrinol Metab 2004; 89:5314-20 (9).

## Unique Patterns of Thyroid Dysfunction in Preterm/Low Birth Weight Infants

### Hypothyroxinemia of Prematurity

**Figure 4:**
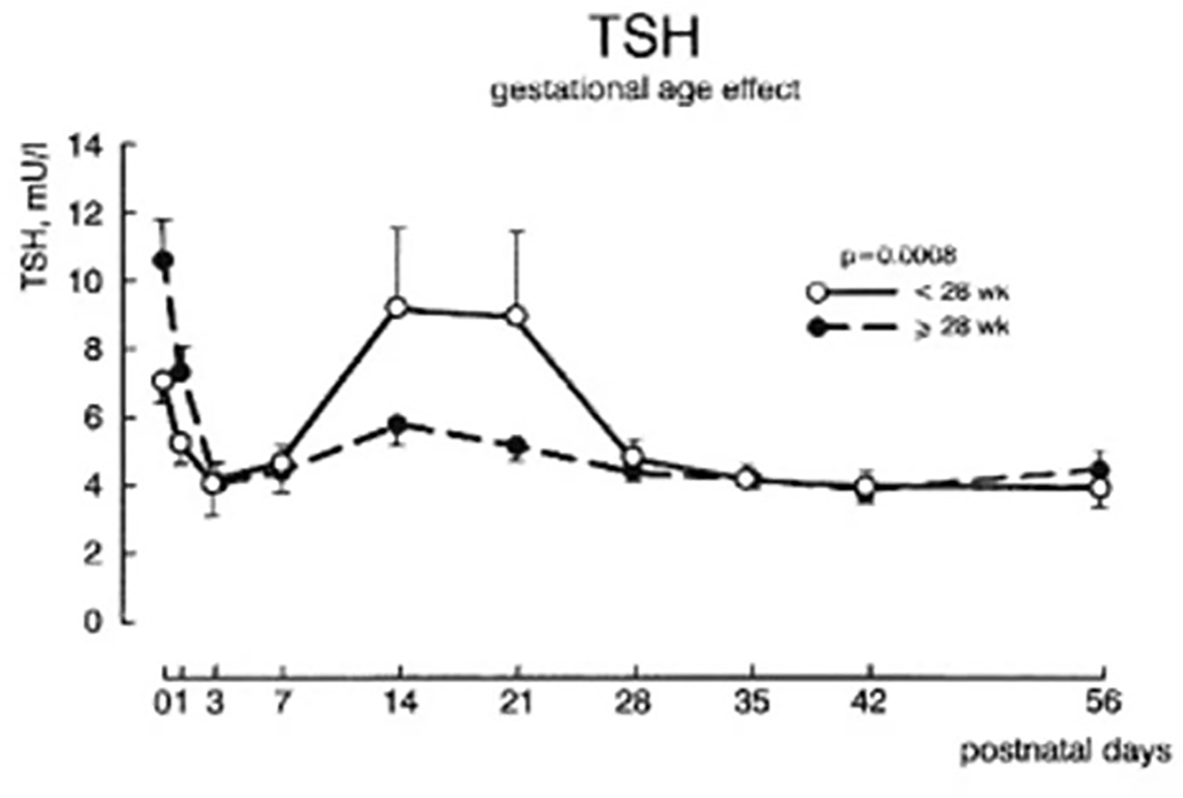
Plasma TSH concentrations ± SEM during the first 8 weeks after birth in infants of 28-30 week gestation and <28 weeks gestation. With permission from van Wassenaer AG, Kok JH, Dekker FW de Vijlder JJ. Thyroid function in very preterm infants: influences of gestational age and disease. Pediatr Res 1997; 42:604-9 (11).

Serum total T4, and to a lesser extent free T4 concentrations are decreased in proportion to the degree of prematurity, generally without elevation of serum TSH levels, findings described as “hypothyroxinemia of prematurity”. In otherwise healthy preterm infants, serum T4 levels gradually rise, such that by 37 weeks gestation they overlap levels seen in term infants (9). In preterm infants born <28 weeks gestation, serum TSH may rise into the 6 to 15 mIU/L range around 2 to 3 weeks of age (11) (**Figure 4**), a transient elevation speculated to play a role in stimulating the immature thyroid gland to normal T4 production, so compensating for the loss of maternal T4 contribution. Common co-morbidities associated with preterm birth, such as respiratory distress syndrome, sepsis, intraventricular hemorrhage, etc. are associated with thyroid function tests typical of NTIS: normal or low total T4, low total T3, elevated reverse T3, and normal to low serum TSH (12). Serum free T4 concentrations measured by the more common analog assay method may be low in this setting, while free T4 measured by the more accurate equilibrium dialysis method tends to be in the normal range (13).

### Delayed TSH Elevation

Some preterm/LBW infants manifest serum TSH concentrations greater than the 15 mIU/L “compensatory” rise described above. The New England NBS program reported an incidence of delayed TSH elevation >20 mUI/mL on the dried blood spot of 1:294 VLBW infants (1000-1500 g) and 1:1878 LBW infants (1501-2500 g), as compared to 1:77,280 in term babies (>2500 g) (14). The delayed TSH elevation occurred at a peak age of 58 days of life (range 11 to 176 days). In even lower birth weight infants, the Wisconsin NBS program reported an incidence of delayed TSH elevation in 1:13 in babies <500 g, 1:28.5 in babies 500 – 1000 g, 1:330 babies 1000-1500 g, and 1:737 babies 1500-2500 g (15). In infants with higher TSH elevation >100 mIU/L, the TSH elevation occurred earlier, ~3 weeks, while those with milder TSH elevation >20 mIU/L occurred later, ~8 weeks of age. Follow-up serum testing has shown that most cases of delayed TSH elevation are transient, particularly if the TSH elevation is mild. In a report from the Rhode Island NBS program, of 19 preterm babies with delayed TSH elevation, three with TSH >50 mIU/L were treated with l-thyroxine, while the other 16 infants monitored without treatment recovered to normal thyroid function between 8 and 58 days of life (16).

### Role of Iodine: Too Little, Too Much

Although universal salt iodization has greatly improved the status of iodine sufficiency (17), worldwide iodine deficiency remains one of the most common preventable causes of intellectual disability (18). Preterm infants are particularly vulnerable to iodine deficiency, a result of a combination of loss of the maternal contribution, relatively low levels of iodine in preterm formulas, and near absence of iodine in most parenteral nutritional preparations (19). A study of iodine balance in 27 to 30 week gestational age infants showed that they were in negative iodine balance for the first two weeks of life, and they did not reach the RDA of 30 mcg/kg/day until 120 days of age (20). Lower iodine intake was correlated with lower serum free T4 and T3 and higher TSH levels. If present, iodine deficiency would be a significant factor contributing to slow recovery from hypothyroxinemia of prematurity.

Exposure to excess iodine has also been demonstrated to cause thyroid dysfunction in preterm infants. Excess iodine results in down regulation of thyroid hormone production (the Wolff-Chaikoff effect), thought to be a mechanism to protect against development of hyperthyroidism. Normal thyroid function is maintained by “escape” from this down regulation; however, preterm infants are slow to escape and so may become hypothyroid. Common sources of iodine exposure include iodinated contrast agents and topical iodine antiseptics. Infants with congenital heart disease (CHD) are frequently exposed to such sources; in a study of 183 infants with CHD, one-quarter manifested elevation of serum TSH levels, ranging from 9 to >100 mIU/mL (21). About half were treated with thyroid hormone, while others recovered to euthyroidism after discontinuation of excess iodine exposure. This study involved primarily term infants; likely the effect of excess iodine would apply to preterm/LBW infants.

### Impact of Preterm Birth/Low Birth Weight on Newborn Screening for Congenital Hypothyroidism

Several NBS programs from Europe and North America report that the incidence of congenital hypothyroidism is higher in preterm infants. As compared to an incidence of approximately 1:1,500 to 1:2,000 in term infants (22, 23), the incidence in preterm infants approximates 1:900 (24). The incidence is even higher in NBS programs that undertake repeat screening in preterm babies, from 1:300 in LBW to 1:50 in VLBW babies (14, 16).

In NBS programs that employ a primary T4-reflex TSH test strategy, preterm/LBW infants make up a higher percentage of cases that fall below the T4 cutoff (typically the 10^th^ percentile), while the screening TSH is not elevated. These NBS programs must then decide whether to follow-up cases with low T4-non-elevated TSH levels. In some programs, lack of an abnormal TSH elevation is the end of screening. However, most NBS programs now collect a 2nd dried blood specimen at 2 to 4 weeks of life in infants born <32 weeks gestation or <1500 g birth weight (25). In the very preterm babies <28 weeks gestation, several NBS programs collect a 3^rd^ dried blood specimen at 6 to 8 weeks of life, which is typically around 37 weeks or term gestation. In the majority of preterm/LBW babies, the screening T4 will reach the range seen in term infants by the 2^nd^ or 3^rd^ specimen (5). Collection of the 2^nd^ and 3^rd^ specimens allows detection of preterm babies with delayed TSH elevation, described above.

A few preterm infants will not normalize total T4 by term gestation; such results could be explained by low binding protein levels or NTIS, although central hypothyroidism cannot be ruled out. In these cases, NBS programs recommend serum thyroid function tests, including TSH, total and free T4. In the presence of low binding proteins or NTIS, serum free T4 is most accurately measured by equilibrium dialysis. A normal free T4 and TSH rule out hypothyroidism, while a low free T4 and low or normal range TSH is compatible with central hypothyroidism, leading to evaluation for other pituitary hormone deficiencies. Such cases in preterm infants are rare, likely with an incidence matching term infants:!:25,000 (26).

In NBS programs that employ a primary TSH test approach, otherwise healthy preterm babies will fall below the dried blood TSH cutoff, typically <25 mIU/L (serum units). As described for T4-reflex TSH test programs, most primary TSH test programs collect a 2nd dried blood specimen at 2 to 4 weeks of life in infants born <32 weeks gestation or <1500 g birth weight, and many collect a 3^rd^ dried blood specimen at 6 to 8 weeks of life in preterm babies born <28 weeks gestation. Collection of the 2^nd^ and 3^rd^ specimens allows detection of preterm babies with delayed TSH elevation, whereas stopping screening with a normal TSH on the 1^st^ test risks missing infants with delayed TSH elevation. Re-evaluation after age 2 to 3 years of age shows that many have transient hypothyroidism (27–29).

### Unique patterns of Thyroid Dysfunction in Preterm/Low Birth Weight Infants: Physiologic or Pathologic?

Preterm/LBW infants manifest clinical features that might be ascribed to thyroid dysfunction, such as temperature instability, apnea with immature pulmonary function and low surfactant levels, bradycardia, slow to feed with sluggish gut motility, edema, hypotonia, and slow growth and development. Several observational studies demonstrate a correlation between low serum T4 levels and these clinical manifestations (30, 31). As these babies typically have low serum T4-non-elevated TSH levels, clinicians face the dilemma as to whether these features are caused by central hypothyroidism and so might improve with thyroid hormone treatment. However, randomized, placebo-controlled trials using either T4 or a combination of T4 and T3 generally do not show an effect on objective measures, such as O2 requirements, incidence of respiratory distress syndrome, requirement for inotropic agents, progression from parenteral to oral feedings, weight gain, length, or head circumference measurements, or on mortality (32–34). A Cochrane Database Review in 2007 also found no benefit of thyroid hormone treatment in this situation (35).

A more compelling question asks whether deficits in neurodevelopment in preterm infants are a consequence of the hypothyroxinemia. Several studies report an increased odds ratio for endpoints such as disabling cerebral palsy (36), reduced attention span (37), vision disturbances (38), and overall lower IQ (39). However, owing to “confounding variables” common to preterm infants, it is difficult to establish a causal relationship with the hypothyroxinemia alone. In a study of preterm infants <1500 g, a comparison of outcome in infants with and without hypothyroxinemia did not find any significant differences in neurodevelopmental, vision or hearing impairment at 5 years of age (40).

What about treatment trials? A randomized, placebo-controlled trial of T4 treatment in 200 infants less than 30 weeks gestational age with multiple assessments of neurodevelopment over time was carried out by the Dutch. Half were treated with l-T4 8 mcg/kg/d, half with placebo for the first 6 weeks of life. In the first assessment at 24 months of age, there were no differences in the Bayley Infant Scales of Mental and Motor Development (41). However, subgroup analysis showed that the Bayley Mental Developmental Index (MDI) score was 18 points higher in the T4-treated group ≤27 weeks gestation, (p<.05), but 10 points lower in the T4-treated group ≥27 weeks gestation (42) (**Table 2**). Re-evaluation at school age (5.7 years) showed that the MDI score had narrowed to 10 points, now not statistically significant (43). Survey of study families at 10 years of age showed that in subjects ≤27 weeks gestation treated with T4, there was a trend toward a lower percentage needing special education, whereas in subjects at 29 weeks gestation, the opposite was true, though only the latter reached statistical significance (**Table 2**). In subjects ≤28 weeks gestation treated with T4, again there was a trend toward better motor outcome as compared to control subjects, but this difference did not reach statistical significance (44). To try and resolve the question of potential benefit in infants born <28 weeks, a more recent trial of thyroid supplementation was undertaken in Amsterdam, Madrid and New York (45). This trial included six treatment arms to investigate different modalities of thyroid hormone administration, combination T4 and T3 treatment, one iodine treatment arm, and one placebo arm. Testing at 36 months of age did not find any differences in neurodevelopmental index scores among the eight groups (**Table 2**). Finally, in a double-blind, randomized, placebo-controlled trial in preterm babies <28 weeks gestation from the United Kingdom, evaluation at 42 months by the Bayley III Mental and Psychomotor Developmental Indices showed statistically better motor, language, and cognitive domains in the group treated with thyroid hormone (46). In summary, the randomized, placebo-controlled trials show mixed results, with potentially some benefit of T4 treatment in infants <28 weeks but potential harm in infants >28 weeks gestation.

**Table 2:** Summary of studies investigating l-T4 treatment v placebo on neurocognitive outcome in preterm infants.

Study (Ref)	Age at Evaluation	Total Group T4 Rx v Placebo	25/26 weeks T4 Rx v Placebo	27 weeks T4 Rx v Placebo	28 weeks T4 Rx v Placebo	29 weeks T4 Rx v Placebo	>27 weeks T4 Rx v Placebo
van Wassenaer (41, 42)	24 mo	92 v 95	93 v 75	90 v 100	97 v 102	92 v 102	92 v 102
	Bayley Mental	P = 0.62	P= 0.01	P = 0.37	P = 0.49	P = 0.36	P = 0.08
	Bayley Psychomotor	92 v 88	80 v 70	81 v 84	98 v 90	86 v 91	90 v 90
		P = 0.39	P = 0.11	P = 0.35	P = 0.29	P = 0.90	P = 0.90
Briet et al. (43)	5.7 years	93.6 ± 16.2 v 95.7 ± 20.4	94.2v 84.7	90 v 92	98 v 96	90.6 v 105.2	
		P = NS	P = 0.11	P = 0.77	P = 0.58	P = 0.01	
van Wassenaer [Questionaire only] (44)	10.5 years	School outcome, motor function, behavior: T4 Rx v placebo P = NS	Percent in Special Ed T4 Rx =10% placebo=28% P=0.07	Motor Im-pairment T4 Rx=12% placebo=28% P = 0.23		Percent in Special Ed T4 Rx=30% placebo=5% P = 0.05	
van Wassenaer (45)	36 months	**Bayley III**		**Bayley III**		**Bayley III**	
		**Cognitive**		**Gross Motor**		**Fine Motor**	
		T4 Rx v placebo		T4 Rx v placebo		T4 Rx v placebo	
		99.2 11.3 v 105.5 ± 12.3		9.3 ± 2.8 v 11.0 ± 2.8		11.1 ± 2.2 v 11.8 ± 2.4	
		P= NS		P=NS		P=NS	
Ng et al. (46)	42 months	**Bayley III**		**Bayley III**		**Bayley III**	
		**Cognitive**		**Motor**		**Language**	
		T4 Rx v placebo		T4 Rx v placebo		T4 Rx v placebo	
		91 ± 10 v 85 ± 13		84 ± 12 v 77 ± 13		92 ± 13 v 83 ± 20	
		P = .045		P = .034		P = .041	

T4 Rx, l-thyroxine treated group.

NS, non-significant.

## Conclusion: Unresolved Questions and Areas of Controversy

Much has been learned over the last few decades about fetal and neonatal thyroid physiology; thyroid function is different in babies born preterm/LBW as compared to term infants. As such, there remain unresolved questions and areas of controversy regarding management of thyroid issues in preterm infants. The following section highlights a few of these areas.

### At What Gestational Age Can the Postnatal HPT Axis Compensate for Loss of Maternal T4 Quickly Enough to Avert Untoward Effects of Thyroid Hormone Deficiency?

A reasonable goal for serum T4 or free T4 levels after birth in preterm babies might be to either match *in utero* concentrations present at a similar gestational age or levels in term infants by 1-2 weeks after birth. Evidence suggests that infants born <28 weeks gestation have serum T4 (and to a lesser extent free T4) concentrations that take up to 4 to 6 weeks to overlap the range seen in term infants. The transient rise in serum TSH to the 6-15 mU/L range between 2 and 4 weeks of age in babies born <28 weeks gestation (but not in babies ≥28 weeks gestation) supports the notion that the thyroid gland in babies born <28 weeks gestation requires increased TSH stimulation to normalize thyroid hormone production. Slow recovery of normal thyroid physiology might impact any organ system, e.g., maturation of lung function, but most importantly it could impact neurodevelopment.

### Should Iodine Supplementation Be Routine in Preterm Infants?

Most preterm infant formulas do not contain enough iodine to allow these babies to meet the RDA of 30 mcg I/kg/d, and many parenteral nutritional preparations lack iodine completely. A recent Cochrane database review did not show benefit of iodine supplementation in preterm infants on morbidity, mortality, or neurodevelopmental outcome (47). Despite this report, given the known effect of iodine to prevent “endemic cretinism”, routine supplementation of preterm formulas and parenteral nutritional preparations would seem important to achieve T4 production matching term infants. Sufficient iodine intake is equally important for nursing mothers. At the same time, care should be taken to avoid exposure to excess iodine.

### Do Infants With “Delayed TSH Elevation” Benefit From Detection and Treatment?

Studies show that the majority of preterm babies with delayed TSH elevation will recover to normal TSH levels without treatment. However, the average age at peak TSH elevation is approximately 8 weeks, with a range from 11 to 176 days of life (14). The TSH elevation likely reflects inadequate thyroid hormone production. Although babies recover to normal thyroid function, are there consequences from low T4 levels, present over several weeks? Few studies have been carried out to address this issue. The study from Rhode Island showed developmental scores were similar to control infants, although the incidence of infants with head circumference <10^th^ percentile was higher in the delayed TSH elevation group (16). It would seem prudent to treat infants with elevated serum TSH and low free T4 levels until recovery to normal thyroid function; since this is difficult to judge without stopping l-thyroxine, most babies are treated until age 2-3 years and then re-evaluated.

### Does T4 Treatment Improve Morbidity/Mortality and Neurodevelopmental Outcome in Preterm Infants <28 Weeks Gestation?

While it is difficult to separate out the effects of co-morbidities from hypothyroxinemia on neurodevelopmental outcome, there are now two randomized, placebo controlled trials in preterm infants <28 weeks gestation that report higher scores in the T4-treated group (42, 46). As noted above, there may be some “physiological” support for this finding, as thyroid function in infants born <28 weeks gestation may be too immature to quickly replace the lost maternal thyroid hormone contribution. That said, the follow-up studies by the Dutch showed that the higher neurodevelopmental scores in the treated group tended to approach the placebo group over time (44). While the more recent trial from Amsterdam, Madrid, and New York did not show benefit of thyroid hormone treatment, the investigators cautioned that “power was insufficient to detect any but very large differences”. With these mixed results, as the saying goes, more randomized controlled trials are needed before such treatment becomes standard of care.

## Author Contributions

SL undertook focused literature review and writing of the manuscript.

## Conflict of Interest

The author declares that the research was conducted in the absence of any commercial or financial relationships that could be construed as a potential conflict of interest.

## Abbreviations

LBW, low birth weight; VLBW, very low birth weight; L-T4, levothyroxine; NBS, newborn screening; NTIS, non-thyroidal illness syndrome; RDA, recommended daily allowance; RT3, reverse T3; TBG, thyroxine binding globulin; T3, triiodothyronine; T4, thyroxine; TSH, thyroid stimulating hormone.
